# Can p503s, p504s and p510s gene expression in peripheral-blood be useful as a marker of prostatic cancer?

**DOI:** 10.1186/1471-2407-5-111

**Published:** 2005-09-05

**Authors:** Maria Rosaria Cardillo, Vincenzo Gentile, Antonio Ceccariello, Laura Giacomelli, Stefano Messinetti, Franco Di Silverio

**Affiliations:** 1 Department of Experimental Medicine and Pathology (Section of Pathologic Anatomy). University "La Sapienza", Viale Regina Elena, 161 Rome, Italy; 2 Department of Urology "U. Bracci", University "La Sapienza", Viale del Policlinico, 156 Rome, Italy; 3 Department of Surgical Science, University "La Sapienza" Viale Regina Elena, 161 Rome, Italy

## Abstract

**Background:**

The aim of the study was to investigate whether p503S, p504S and p510S gene expression in peripheral-blood be useful as a diagnostic or prognostic marker of prostatic cancer.

**Methods:**

Circulating cells were identified by reverse transcription-polymerase chain reaction (RT-PCR) to detect p503S, p504S and p510S mRNA in peripheral blood (PB) from 11 patients with treated prostatic carcinoma (CaP), 11 with newly-diagnosed untreated CaP and 20 with benign prostatic hyperplasia (BPH) (controls).

**Results:**

RT-PCR amplified P503S in 7 of 11 untreated and 2 of 11 treated patients with CaP and 5 of 20 with BPH; p504S in 7 of 11 untreated and in 9 of 11 treated patients with CaP and 11 of 20 with BPH; whereas it amplified p510S in all subjects with CaP and in 15 of 20 with BPH.

**Conclusion:**

These findings suggest that the investigated genes are poorly specific and probably of little use as diagnostic or prognostic prostatic markers in peripheral blood for monitoring disease progression and recurrence.

## Background

Microarray approaches have identified three prostate tissue and cancer-prostate-specific genes: p503S, a 241-amino acid protein that encodes human tetraspan NET-1, a member of the tetraspanin/TM4SF family, involved in cancer metastasis) [1], P504S, also referred to as the AMACR gene, encodes human α-methylacyl-CoA racemase, a 382-amino acid protein involved in the conversion of R-stereoisomers of branched-chain fatty acids to S-stereoisomers [2-6] and p510S, identified as the human ABC transporter MOAT-B [7]. All these genes are overexpressed in prostate tumor or normal prostate tissue or both and are considered clinical biomarkers of prostate cancer (CaP)[8]. The immune response against AMACR has been used as a serum biomarker for CaP [9,10] and the quantification of AMACR transcripts in prostatic secretions shown to be predictive of CaP [11].

Attempts to detect AMACR in circulation have been disappointing and no molecular studies have yet sought p503S and p510S circulating cells by reverse transcriptase-polymerase chain reaction (RT-PCR) assay of peripheral blood (PB) from patients with CaP.

The aim of this study was to investigate whether p503S, p504S and p510S genes are diagnostic or prognostic prostate-specific markers for monitoring disease progression and recurrence. Extending our previous research investigating biomarkers that are clinically important to distinguish patients with prostate cancer at risk for early relapse from patients in clinical remission [12-14], in this study, we analyzed by RT-PCR assays p503S, p504S and p510S gene markers in PB from 42 patients with treated or untreated CaP and with benign prostatic hyperplasia (BPH) (controls).

## Methods

### Patients and control tissue specimens

The 42 participants included in this study were consecutively selected from patients treated at the Department of Urology "U. Bracci", University of Rome "La Sapienza". We studied three clinical groups. The first group comprised 11 patients with CaP (mean age at diagnosis 68.9 years; range 45–89) who underwent androgen ablation (AA) followed by retropubic radical prostatectomy (RP) and bilateral lymphadenectomy between January 1997 and December 1999. Tumors were graded using the Gleason system for histologic grading of CaP: two tumors were Gleason score sum 4, 7 Gleason sum 7 and 2 Gleason sum 8. Tumors were then evaluated clinico-pathologically using the tumor-node metastatic (TNM) staging system 2002: 7 tumors were organ-confined diseases (pT2) and 4 were extra-prostatic diseases (pT3). Follow-up after treatment ranged from 3–84 months (mean, 27 months). At the time of venipuncture for PB, we distinguished two subgroups: patients who responded to the first 24 months of treatment without clinical progression; and AA resistant patients who received neoadjuvant androgen deprivation therapy because recurrent disease developed within 24 months after RP. We defined recurrence by serum PSA levels, transrectal ultrasound (TRUS) and computed tomographic (CT) scan. Men were considered disease free after RP if they had serum PSA levels less than 0.1 ng/ml. The second subgroup comprised 11 patients with elevated serum PSA levels (more than 4.0 ng/ml), who had received a diagnosis of CaP based on TRUS-guided-prostate biopsy (1 tumor was Gleason sum 6 and 10 were Gleason sum 7). All 11 patients had organ-confined disease: 4 were pT1 and 7, pT2. In patients treated for CaP, PB samples were obtained 27 to 84 months (mean 55 months) after treatment ended; in patients who had received a diagnosis of CaP based on clinical and biochemical evidence of malignancy, PB samples were obtained before biopsy and before treatment began. The third group (control group) comprised 20 men 50 years of age or older (mean age 58.7 years; range 45–79) with no known malignancy, with a histologic diagnosis of BPH in tissue obtained by fine-needle biopsy of the prostatic glands and on conventional clinical data (serum PSA levels less than 4.0 ng/ml, and digital rectal examination and TRUS negative for CaP). The control group also included a PB sample from a patient who underwent radical cystectomy for bladder cancer (G3T1) and 6 neoplastic and non neoplastic prostatic tissues; 6 tissues from various organs (adrenal gland, heart, myometrium and uterine cervix); and 6 prostatic (SV48, LS147D, SCOV 3 and MCF7) and non prostatic cell lines (PC3 and LNCaP).

### Reverse-transcription polymerase-chain-reaction (RT-PCR)

Total RNA was extracted from whole blood cells and cell lines using the technique previously described [12-15]. Total RNA from frozen tissue was extracted using the protocol developed by Ambion, and detailed in the RNA isolation Kit (Ambion Inc., U.S.A). cDNA (250 ng) from each sample was amplified by PCR using the following primers: sense, 5'TGCCCTCGTGACGTTCTTCT3' and antisense 5'TCTTTCTTGATGGCAGGCACTAC3' of P503, 136 bp (GeneBank accession number AF065388 (16) using 35 cycles (94°C for 30", 60°C for 30" and 72°C for 60 sec); sense 5'AAATGGTTATCATTAGGGCTTTTGA3' and antisense 5'TTCCTTTTTCACTAGAACCCATTCA3' of P504S, 149 bp (GeneBank accession number 4204097 [17] using 35 cycles (94°C for 30", 55°C for 30" and 72°C for 60 sec); sense 5'TTGAACAGCTACTACGGTCAATGTATT3'and antisense 5' GCAGAGAGCAACCGATGTTTT3' of P510S, 96 bp (GeneBank accession number AF071202 [18] using 35 cycles (94°C for 30", 57°C for 30" and 72°C for 60 sec) by Platinum Taq DNA polymerase (Gibco BRL), according to the manufacturer's protocols. The integrity of RNA was checked in a preliminary PCR reaction for a human β-actin 743 base-pair (bp) fragment [19]. Each RT-PCR experiment included a non retrotranscribed sample as a negative control and c-DNA from LNCaP cell lines as a positive control. PCR-amplified P510S sequence specificity was checked by direct sequencing of the amplified product by the automated DNA sequencer A.L.F. (Pharmacia, Freiburg, Germany). The sequencing reaction was run on an Abby Applied 377 Sequencer (Applied Biosystems). Band identity was verified by comparison with the genomic and cDNA P510S sequences at the GeneBank using the BLAST program.

## Results

**Figure 1:**
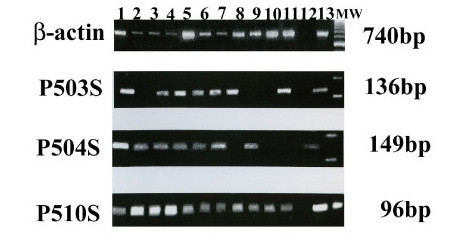
Analysis of gene expression levels of p503S, p504S and p510S by RT-PCR using β-actin yielded a 740 base-pair band as internal control, in whole blood from 11 patients after radical prostatectomy and androgen ablation (lanes 1 to 11); negative control, no RNA (lane 12); positive control (LNCaP cell line) (lane13); MW (molecular weight).

RT- PCR analysis amplified p5103S-, p504S- and p510S-mRNA in PB from nearly all patients who underwent AA and RP, who had recurrent disease and p503S and p504S in only 2 (40%) of the 5 patients without recurrence (Fig. 1; Table 1). Comparing the RT-PCR findings with the histological Gleason scores we found p503S- and p504S-positive cells in a higher percentage of patients with higher-grade Gleason scores than lower-grade scores. P503S were more often expressed in extraprostatic disease (pT3N1 and pT3N2) and p504S in organ-confined disease (pT2N0). The p510S gene was amplified in all patients regardless of the clinical subgroup (Table 2).

**Figure 2:**
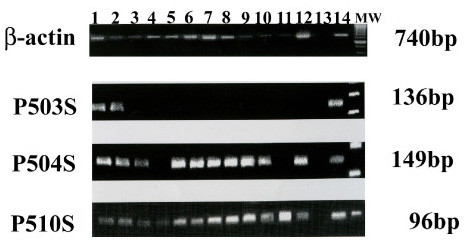
Analysis of gene expression levels of p503S, p504S and p510S by RT-PCR using β-actin yielded a 740 base-pair band as internal control, in whole blood from 11 patients with CaP after biopsy (lanes 1 to 11) and one patient with transitional cell carcinoma of the bladder (lane 12) as control. Negative control (no RNA) (lane 13); positive control (LNCaP cell line) (lane 14); MW (molecular weight).

Two samples from 11 untreated patients with biopsy-proven CaP contained p503S mRNA. This gene was expressed significantly less frequently than p504S and p510S (18% vs 81 and 100% (P <.01, by chi-square test) (Fig. 2; Table 1). PB from the control patient with non prostate malignancies, who underwent radical cystectomy for transitional-cell carcinoma (G3T1), contained p504S- and p510S-but no p503S-positive cells (Fig. 2, lane 12). Nearly all untreated patients with Gleason score 6–7 tumors and organ-confined disease had p504S- and p510S-positive cells, whereas only 2 of the 11 patients had p503S-positive cells. p503S-positive cells were detectable in patients with stage pT2 and undetectable in those with stage pT1 disease. p510S-positive cells were expressed in similar percentages of patients with pT1 and pT2 disease (Table 2).

**Figure 3:**
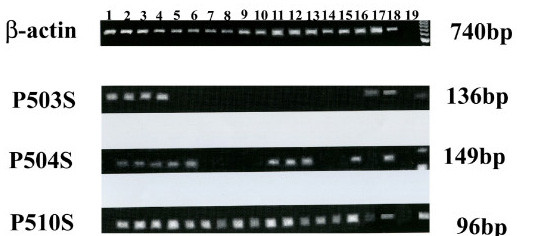
Analysis of gene expression levels of p503S, p504S and p510S by RT-PCR using β-actin yielded a 740 base-pair band as internal control in whole blood from 17 of 20 patients with benign prostatic disease (BPH).

**Figure 4:**
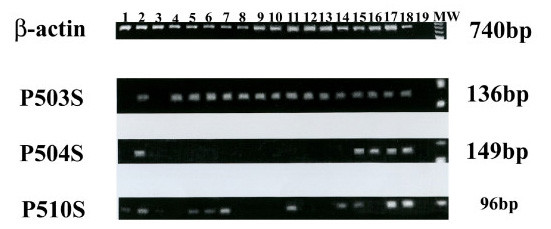
Analysis of gene expression levels of p503S, p504S and p510S by RT-PCR using β-actin yielded a 740 base-pair band as internal control in frozen benign (lanes 1, 3, and 6) and neoplastic (lanes 2, 4, and 5) prostatic tissue; non prostatic tissue (adrenal gland (lane 7); heart (lanes 8 to 10); myometrium (lane 11); uterine cervix (lane 12) and human cell lines: SV48 (lane 13); LS147D (lane 14); SCOV 3 (lane 15); MCF7 (lane 16); PC3 (lane 17); and LNCaP (lane 18). Negative control no RNA (lane 19); MW (molecular weight).

**Figure 5:**
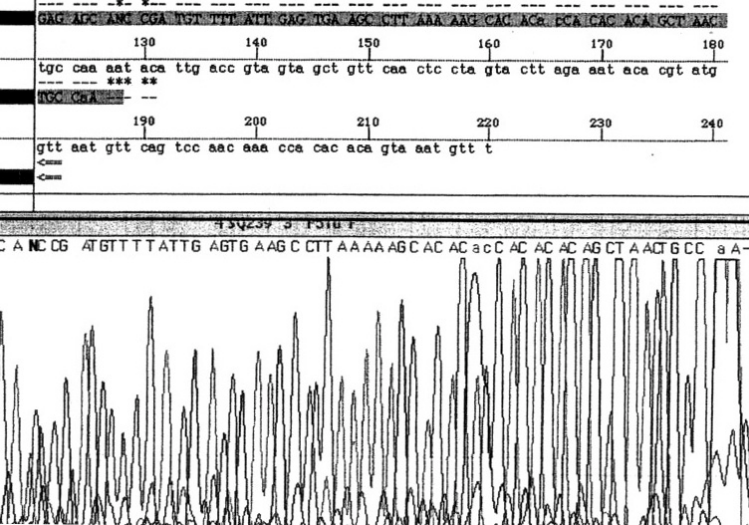
Nucleotide sequence of P510S cDNA.

In PB samples from the control group (BPH), PCR amplified more p504S- and p510S- than p503S-positive cells (55 and 75% vs 25%) (Fig. 3). Nearly all the prostatic and non prostatic-tissues and cell lines used as controls expressed p503S-, p504S-, and p510S-mRNA (Table 1) (Fig. 4). DNA sequencing of the amplified 96 bp fragment confirmed the specificity of these primers for the p510S gene (Fig. 5).

## Discussion

These findings argue against the usefulness of the p503S, p504S and p510S genes as diagnostic and probably against their usefulness as prognostic prostate-specific markers for monitoring disease progression and recurrence in PB from patients with CaP. Our findings obtained by RT-PCR assay contrast with Zelie et al [11] et al who found the RT-PCR-AMACR test non-invasive and useful in predicting the presence of CaP in prostatic secretions. When we compared our findings for p503S, p504S and p510S with our earlier study investigating prostatic specific antigen (PSA) [3], as potential markers for prostate tumors, all these three genes were more frequently expressed than PSA in PB from patients with treated CaP (63%, 63% and 100% vs 18%) and untreated CaP (18%, 81% and 100% vs 9%) and in PB from BPH (25%, 55% and 75% vs 0). In benign and malignant prostate tissue, p510S was more strongly expressed than PSA, or p503S and p504S (100% vs 66% vs 66%), whereas in prostatic cell lines, PSA and p503S, p504S and p510S were similarly expressed (100%). In non prostatic tissue and cell lines, PSA was not expressed; in contrast, in non prostatic samples and cell lines, p503S was more expressed than p510S and p504S (100% vs 33% vs 16%) and cell lines (100% vs 50% vs 50%). Hence, the increased expression of PSA mRNA in prostate tissue suggests that PSA is more specific than p503S, p504S and p510S as a marker of cancer progression and dissemination.

The ubiquitous presence of p503S, p504S and p510S in PB of patients with BPH, in non prostatic tissue and cell lines therefore argues against their potential value as biomarkers of prostate disease. Compared with PSA [13], none of the three newly-identified putative CaP markers we studied were sufficiently sensitive or specific to distinguish patients in complete clinical remission from patients with occult metastatic disease at high-risk for relapse. The numerous false-positive findings in our assay and the reported presence of p503S, p504S and p510S in normal hematopoietic tissues also from patients without prostate malignancies [20], could depend on illegitimate expression of gene transcripts. This question awaits an answer from studies designed to investigate the whole p503S, p504S and p510S gene sequence. For RT-PCR analysis of PB cells in this study we used primers for three new putative genes (p503S, p504S, and p510S) whose prostate cancer specificity has already been confirmed by Northern blot, real-time PCR and immunohistochemical assay on prostatic tissue [3-8]. Although AMACR may be potentially useful as a tissue biomarker for prostate cancer [3,4], the limitations of this assay make it impossible to use an autoimmune response against AMACR as a means of detecting this antigen in the serum [10]. Nor did a novel approach to predict the presence of CaP from prostatic secretions prove predictive of CaP [9]. Whether these new markers have potential clinical utility as sensitive and specific indicators of CaP progression therefore warrants further research using quantitative RT-PCR technique to study the entire gene sequences.

## Conclusion

We provide the first evidence of the ubiquitous presence of p503S, p504S and p510S in PB of patients with BPH, in non prostatic tissue and cell lines. These findings suggest that the investigated genes are poorly specific and probably of little use as diagnostic or prognostic prostatic markers in peripheral blood for monitoring disease progression and recurrence.

## List of abbreviations used

RT-PCR – reverse transcription-polymerase chain reaction

CaP – prostatic carcinoma

BPH – benign prostatic hyperplasia

AMACR – α-methylacyl-CoA racemase

PB – peripheral blood

AA – androgen ablation

RP – radical prostatectomy

TRUS – transrectal ultrasound

CT – computed tomographic

TNM – tumor-node metastatic

PSA – prostatic specific antigen

## Competing interests

The author(s) declare that they have no competing interests

## Authors' contributions

MRC provided input into the design of these studies, assistance with development of the assays used for these molecular genetic studies in the laboratory of Uropathology at Department of Experimental Medicine, Rome "La Sapienza" University, data analysis and writing of the manuscript.

AC provide assistance with patients diagnoses undergone TRUS-guided-prostate biopsy and collection of specimens and clinical date.

VG provide assistance with patients diagnoses and collection of specimens and clinical data, and supervised all aspect of the work performed for this paper in the Department of Urology, Rome "La Sapienza" University.

LG provided collection of specimens and clinical data, and assisted with data analysis.

SM provided assistance with patient diagnoses and with data analysis.

FDS provided assistence with patients undergone surgery, collection of specimens and clinical data, and supervised all aspect of the work performed for this paper in the Department of Urology, Rome "La Sapienza" University.

## Pre-publication history

The pre-publication history for this paper can be accessed here:



## Figures and Tables

**Table 1:** RT-PCR assay in peripheral blood, in different tissues and cell lines.

SAMPLES	RT-PCR	P503S	P504S	P510S
PB		No (%)	No (%)	No (%)

group 1				
AA and RP (n = 11)	positive	7 (63.63)	7 (63.63)	11 (100)
	negative	4(36.36)	4(36.36)	0
group 2				
Pca in prostate biopsy (n = 11)	positive	2 (18.18)	9 (81.81)	11 (100)
	negative	9 (81.81)	2 (18.18)	0
group 3				
BPH in prostate biopsy (n = 20)	positive	5 (25)	11 (55)	15 (75)
	negative	15 (75)	9 (45)	5 (25)
				
Frozen tissues				
				
Neoplastic and non neoplastic	positive	4 (66.66)	4 (66.66)	5 (83.83)
prostate tissues (n = 6)	negative	2 (33.33)	2 (33.33)	1 (16.16)
				
Non prostatic tissues (n = 6)	positive	6 (100)	1(16.66)	2 (33,33)
	negative	0	5 (83.33)	4 (66.66)
				
Cell lines (n = 6)				
				
prostatic (n = 2)	positive	2 (100)	2 (100)	2 (100)
	negative	0	0	0
				
non prostatic (n = 4)	positive	4 (100)	2 (50)	2 (50)
	negative	0	2 (50)	2 (50)

PB = peripheral blood

RT = radical prostatectomy

AA = androgen ablation

**Table 2:** p503S, p504S and p510S positive cells in peripheral blood of patients with prostate cancer (CaP) in relation to Gleason score and TNM stage.

	P503S+	P504S+	P510S+
**Group 1 (treated CaP)**	No	No	No
Total (n = 11)	patients (%)	patients (%)	patients (%)
subtype 1 without recurrence (n = 5)	2 (40)	2 (40)	5 (100)
subtype 2 with recurrence (n = 6)	5 (83)	5 (83)	6 (100)
			
*Gleason score*			
4–7 (n = 9)	4 (44.44)	6 (66.66)	9 (100)
8 (n = 2)	2 (100)	2 (100)	2 (100)
*TNM*			
Organ-confined disease (n = 7)			
T2 N0 M0 (n = 7)	4 (57.14)	5 (71.42)	7 (100)
Extraprostatic disease (n = 4)			
T3 N0 M0 (n = 2)	1 (50)	1 (50)	2 (100)
T3 N1M0 and T3 N2M0 (n = 2)	2 (100)	1 (50)	2 (100)
			
**Group 2 (untreated CaP)**			
Total (n = 11)			
*Gleason score*			
6–7 (n = 11)	2 (18.18)	9 (81.81)	11 (100)
*TNM*			
Organ-confined disease (n = 11)			
T1 (n = 4)	0	3 (75	4 (100)
T2 (n = 7)	2 (28.6)	6 (85.7)	7 (100)
